# Ultrasound‐assisted extraction of oil from hempseed (*Cannabis sativa* L.): Part 1

**DOI:** 10.1002/jsfa.11404

**Published:** 2021-07-18

**Authors:** Marilena Esposito, Laura Piazza

**Affiliations:** ^1^ Department of Environmental Science and Policy Università degli Studi di Milano Milan Italy

**Keywords:** ultrasound‐assisted extraction, hempseed press cake, hempseed oil, oil quality

## Abstract

**BACKGROUND:**

Ultrasound‐assisted extraction of the intermediate product from the mechanical expression of hemp (*Cannabis sativa* L.) seed oil was investigated to improve the overall expression yield without compromising oil quality. Complementary ultrasound technology was used as an out‐of‐line treatment carried out at 20 kHz frequency and optimized with respect to amplitude (80 and 152 μm), sonication time (2, 10, 20 min) and to the hemp paste properties, in particular its particle size and hydration, which drive the compressibility of the press cake.

**RESULTS:**

Under the conditions evaluated, the optimal ultrasound treatment was found to be the one applied on the hydrated press cake for 2 min at 152 μm, which resulted in an oil yield of 13.4%, with an increase in extraction efficiency equal to 73% with respect to the control (untreated press cake). Sonication had a positive effect on the press cake texture and on the extracted oil antioxidant activity. Soaked samples treated for 2 min at 152 μm yielded the lowest hardness. Oil recovered from soaked samples treated at 80 μm and 152 μm ultrasound for 2 min had the highest antioxidant capacity.

**CONCLUSIONS:**

The technological results gathered in the present investigation are preliminary to the design and engineering of scaled‐up equipment that combines the mechanical screw expression and the in‐line ultrasound unit. © 2021 The Authors. *Journal of The Science of Food and Agriculture* published by John Wiley & Sons Ltd on behalf of Society of Chemical Industry.

## INTRODUCTION

The nutritional quality of edible oils derived from vegetable seeds has become a competitive factor on the market for acceptability of the product by the consumer. A peculiar compositional fact regarding the non‐drug hemp (*Cannabis sativa* L.) seeds is its high oil content. Specifically, oil is rich in polyunsaturated fatty acids, which represent approximatively 70–80% of total fatty acids. Linoleic acid (LA; 18:2, omega‐6) and linolenic acid (ALA; 18:3, omega‐6) are the most represented unsaturated acids in an ideal ratio (3:1) for human health. This balance is practically unique among the common plant oils. Moreover, hempseed oil contains a large amount of antioxidants such as tocopherols and carotenes, resulting in a worthwhile nutritional value.^1^, ^2^ Hempseed oil is conventionally obtained by mechanical screw expression,^3^ whose advantages include low cost, recognized quality of oil and opportunities to reuse the expression by product (the expression cake or ‘meal’). However, this method usually is relatively inefficient in terms of oil yield, leaving about 8–14% of available oil in the cake.^4^, ^5^ On the other hand, the n‐hexane solvent extraction method would be more efficient because it results in the highest yield (95%), but it would require a longer extraction time and an extra refining step because of solvent residues in the final product.^6^

There is a growing interest for ultrasound inclusion in extraction of oil (soybean, almond, sunflower), proteins and bioactive compounds (polyphenols, polysaccharides, anthocyanins) from different sources.^7^, ^8^, ^9^, ^10^ All authors agree that ultrasonic assisted extraction (UAE) advantageously improves conventional oil extraction technology because of high extraction yield, shorter extraction time and low energy demand.^11^, ^12^, ^13^ During the UAE of vegetable materials, the result of the cavitation phenomena is an enhanced material transfer from inside the cells to the external environmental. The effective frequency range for this technique, as reported in the scientific literature, is 20–100 kHz.^12^, ^14^ Recent publications show the advantages in terms of efficiency and reduction extraction time for the UAE extraction of extra virgin olive oil.^15^, ^16^, ^17^ Clodoveo *et al*.^15^ underline that the UAE process of virgin olive oil showed a reduction of malaxation with an overall enhanced quality of olive oil. Massa *et al*.^18^ investigated the UAE of oil from the seeds/peel mixture of pumpkin using ethanol as solvent to reduce negative impacts on the environmental, since it is considered to be a safe solvent and comes from renewable sources. In this study, treatment in an ultrasonic bath was performed at 25 kHz frequency and 165 W power, and the influence of temperature, time and solvent‐to‐solid ratio was investigated. It was found that the highest oil yield was reached at 90 min and 75 °C, with an optimum ratio of 6 mL g^−1^. On the other hand, in a study conducted by Santos *et al*.^19^ ultrasonic apparatus with a probe was used in the oil extraction from favela seeds, and the results showed that the efficiency of oil extraction as well as the highest yields in bioactive compounds were obtained below 60 °C for 5 min and a solvent‐to solid ratio of 15 mL g^−1^.

Regarding hempseeds, Da Porto *et al*.^6^ observed that ultrasound pretreatment of hempseeds using a probe with 20 kHz working frequency and 200 W amplitude for 10 min increased the oil extraction yield with respect to the solvent extraction.

Based on current knowledge, the extraction efficiency depends on different process parameters such as sonication time and temperature, sonication amplitude and particle size. Therefore, the optimization of UAE operating conditions would allow improved oil extraction and guaranteed oil quality.

A study is presented focused on the properties of the UAE of hemp press cake, also known as ‘meal’, in order to improve oil extraction efficiency. Promoting the yield of oil extraction from hempseeds has an economic advantage both for the considerable added value of commercial hemp oil and because the residue of extraction, the semi‐exhausted cake, typically containing a percentage of residual oil in the cake of around 10%, shows a more extended chemical stability if the residual oil inside drops further. Toward this goal, a process intensification revises the implementation of an ultrasound‐assisted mechanical extraction process. Investigations are preliminary to a scaled‐up mechanical oil expression combined with in‐line ultrasound treatment of the oleaginous material.

Different operating conditions will be evaluated in the study for the optimization of ultrasound treatment of the semi‐exhausted cake that will be pulled out close to the end of the mechanical extractor. In particular, ultrasound amplitude and ultrasound treatment time, cake particles size and moisture content will be tested for a maximum oil yield. The quality of the expressed oil has to be guaranteed whatever is the process used for oil extraction. Main chemical and physicochemical indices of oil quality will be therefore quantified.

## MATERIAL AND METHODS

The chemicals used, which included n‐hexane, methanol, Trolox (6‐hydroxy‐2,5,7,8‐tetramethylchroman‐2‐carboxylic acid), 1,1‐diphenyl‐2‐picrylhydrazyl (DPPH), were of analytical reagent grade and were purchased from Sigma‐Aldrich Co., Milan, Italy. Hemp (*Cannabis sativa* L. var. Futura 75) milled paste, or cake or ‘meal’, was kindly supplied by Next Farm Company (Bagnolo Cremasco, Italy) and was pulled out close to the end of the mechanical extractor.

### Mechanical pressing for oil expression

**Figure 1:**
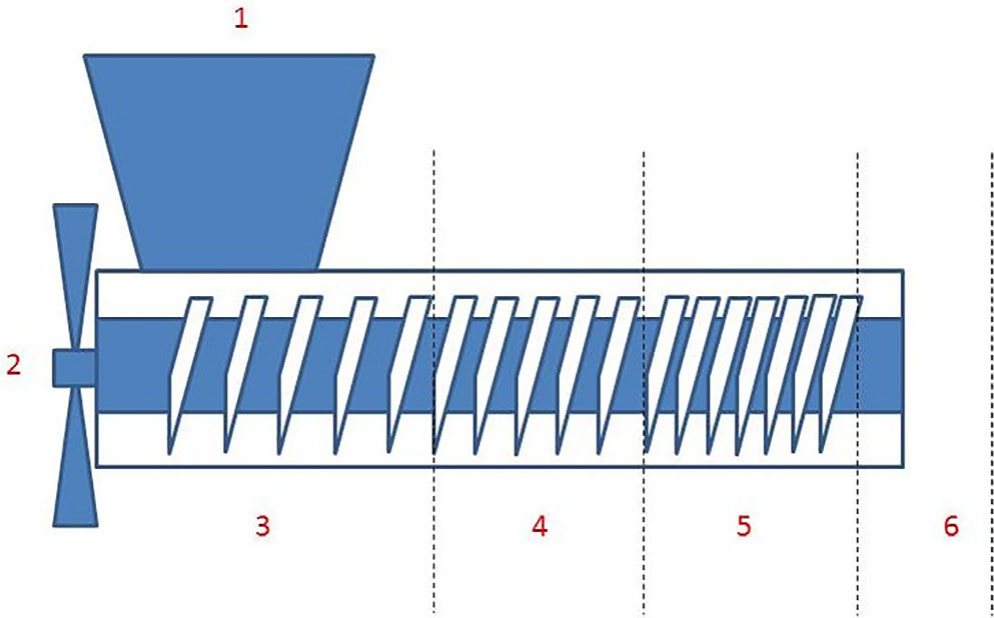
Scheme of hemp oil processing line (image courtesy of Next Farm, Bagnolo Cremasco, (CR), Italy): 1, solid feed; 2, electric motor and inverter; 3, first oil expression section; 4, second oil expression section; 5, exhausted cake discharge section; 6, die and discharge of residual cake on the screw.

Mechanical oil expression (also known as pressing) was conducted in a screw press by the Next Farm company (Bagnolo Cremasco, Italy), where the pressing force is created by a helical screw rotating in a barrel The seeds are driven by the screw and the barrel ensures the progression of the seeds by preventing the cake to ‘turn en masse’. Pressing is the main step of oil extraction during which most of the oil is extracted from the seeds and advance along the length of the press (Fig. 1). The barrel temperature was monitored, ranging from 35 to 39 °C.

The expressed oil is collected separately in the first and second sections of the screw extractor (sections 3 and 4 in Fig. 1). The final extraction yield accounts for the sum of the two fractions. According to the manufacturer concerned, different oil yields are announced depending on the hempseed harvesting quality.

For the purpose of this work, the cake to be used for technological and analytical investigations was picked up at section 2 of the screw (Fig. 1). The cake was collected and quickly packaged under mild vacuum to avoid oil oxidation. Transfer to the laboratory was made in refrigerated boxes.

### Quantification of the expressible oil

Extraction of the residual oil in cakes was carried out as reported by Zhang *et al*.,^12^ with slight modification. Press cake (10 g) was mixed with n‐hexane (1:10) in a flask with agitation for 3 h. The flask was then placed in a water bath with a controlled temperature of 35 °C. The extracts were filtered through Whatman filter paper and then collected and concentrated with a Rotavapor (Laborota 4010 digital rotary evaporator, Heidolph Instruments GmbH & Co. KG, Schwabach, Germany) to acquire the oil. The latter was vacuum dried to remove the residual solvent.

### Ultrasound processing

An ultrasonic processor with a flat‐tip probe (ultrasonic liquid processor, Fisher Scientific, Waltham, MA, USA) at a frequency of 20 Hz and ultrasonic power 500 W was used for ultrasound‐assisted extraction of oil from the press cake. Ultrasound treatments varied for application time (2, 10 and 20 min) and amplitude (80 and 152 μm). During the treatment, the rise in temperature was controlled by placing the flask with the meal in a beaker containing ice water, and the overall temperature of the sample was maintained at around 35 °C ± 0.3 °C. The residual extractable oil in cakes was quantified as previously described and the oil yield was calculated according to Eqn (1):

(1) $$\text{Oil yield}\;\left(\%\right)=\left(q_{o}/q_{s}\right)\times 100$$

where *q*_o_ is the mass of oil obtained (g) and *q*_s_ is the initial mass of seeds (g) used in the experiment. All the experiments were performed in triplicate.

### Radical scavenging activity: DPPH assay

The ability of oil extracted from press cake to scavenge DPPH free radicals was assessed using the method described by Lin *et al*.^20^ with some modifications. Briefly, 0.1 mL extract oil was mixed with 2.9 mL methanol solution of 0.1 mmol L^−1^ DPPH, shaken vigorously and incubated in darkness at room temperature for 60 min. The absorbance values of DPPH‐added oil samples were recorded at 517 nm using a UV–visible spectrophotometer (Jasco V‐650, Tokyo, Japan). The inhibition percentage of DPPH was determined using the following equation:

(2) $$\text{DPPH inhibition}\;\left(\%\right)=100\left(\mathrm{Abs}\;\text{DPPH}-\mathrm{Abs}\;\text{sample}\right)/\mathrm{Abs}\;\text{DPPH}$$

where Abs DPPH is the absorbance value at 517 nm of the methanol solution of DPPH and Abs sample is the absorbance value at 517 nm for the sample.

The oxidation stability of oil was also expressed as millimoles of Trolox equivalents per millilitre of oil through a calibration curve of Trolox standard solution in methanol. Each sample was assayed in triplicate.

### Free fatty acidity determination

According to the method reported by Rapa *et al*,^21^ an aliquot of 10 g oil was dissolved 100 mL diethyl ether/ethanol (60/40) mixture. The free fatty acids were titrated, under shaking, with 0.1 mol L^−1^ NaOH until the phenolphthalein indicator turned pink. Acidity was expressed according to the following equation:

(3) $$\text{Acidity}\;\left(\%\right)=\left(\mathrm{VCM}\right)/\left(10m\right)$$

where *V* is the volume of the titrated solution of NaOH used, *C* is the concentration of NaOH, *M* is the molar weight of the acid used for the expression of the result (oleic acid = 282), and *m* is the weight of the substance analysed.

### Colour measurements

Objective colour parameters of oils were measured by means of a spectrophotometer (CM‐2600, Minolta, Japan) using the CIE L* a* b* scale and the instrument was calibrated with a white calibration plate. In more detail, a* is positive for reddish colours and negative for greenish ones. The coordinate b* is positive for yellowish colours and negative for bluish ones. L* is an estimation of luminosity and allows to any given colour to be regarded as equivalent to a member of the grey scale, between black (L* = 0) and white (L* = 100). The oil samples, diluted in isooctane 1:6, were placed in 13 mL cuvettes (10 mm path length). Data collected included lightness values (L*), redness values (a*) and yellowness values (b*), and the total colour difference (Δ*E*) was calculated using the following equation:

(4) $$\Delta E=\surd {\left(L{*}_{0}-L*\right)}^{2}+{\left(a{*}_{0}-a*\right)}^{2}+{\left(c{*}_{0}-b*\right)}^{2}$$

where L*_0_, a*_0_ and b*_0_ were the values of the control, and L*, a* and b* were the values of the treated sample.

### Mechanical properties of the cake

Mechanical expression of fluids from biological materials depends on the compressibility/incompressibility of the cake. In order to quantify the packing arrangement in the cake, compression tests were performed at room temperature on untreated and selected ultrasound‐treated cake by means of a texture analyser (TA‐XT2, Stable Microsystems, Godalming, UK), equipped with an Ottawa cell, with circular openings at the bottom and a wire plate piston (A/WIR) geometry hosting 50 g cake, using a 50 kg load cell. Tests were carried out until compression was equal to 40% of the original height. The following experimental conditions were adopted: pre‐test speed 1 mm s^−1^; test speed 2 mm s^−1^; post‐test speed 10 mm s^−1^; trigger force 1 kg. Compression work (N mm), expressed by the area under the force–distance curve, and hardness (N), quantified as the maximum compression force, were evaluated. All the tests were performed in duplicate.

### Statistical analysis

All analyses were performed in triplicate and the results were recorded as mean ± standard deviation. Origin software (OriginLab Corporation, Wellesley Hills, MA, USA) was used for statistical analysis by one‐way ANOVA with Tukey's HSD, with the level of significance set at *P* ≤ 0.05.

## RESULTS AND DISCUSSION

### Yield optimization in oil extraction

Screw pressing of oleaginous vegetable material is the most popular oil expression method as the process is simple, continuous, flexible and safe. In order to increase the extraction yield, process intensification through UAE of oils has been suggested for oleaginous materials.^22^ Shear stress conditions and pressure fields applied to the oilseeds along the mechanical press reactor can affect the oil extraction yield, depending on the overall liquid‐to‐mass ratio of the paste. Moreover, the solid size will affect paste compressibility, which drives the expression of liquids from the liquid‐containing solids.

Trials were planned in this work to improve the extraction yield, by testing both different ultrasound operating conditions and some material properties of the semi‐exhausted paste. The press cake, a heterogeneous high‐density dispersion in which an oil‐in‐water emulsion imbibes the solid fraction, was collected at the end of the second extraction zone in the screw press; thus it was partially compressed and de‐oiled. Residual oil and moisture content in the press cake samples were determined, resulting in 0.73 ± 0.14 g kg^−1^ and 0.56 ± 0.1 g kg^−1^, respectively.

The following operating conditions for the out‐of‐line sonication were adopted: 500 W ultrasonic power and 20 kHz frequency ultrasonic waves. The temperature of the sample was kept constant at around 35 °C. In order to maximize the oil yield, ultrasound extraction was investigated at two different amplitudes (80 and 152 μm) for various sonication times (2, 10, 20 min).

The oilseed cake is assimilated to a porous medium.^23^ In screw pressing, the oiled solid is propelled by the screw and compressed by gradually reducing the volume available to the material.^24^ The process is pressure driven and involves the flow of oil through the porous bed. Pressure is required to overcome the resistance of the solid matrix to deformation and compaction, to disrupt the cells and to compensate for the pressure drop arising from the flow of the fluid through the solid.^25^ The compressibility of the solids, accounting for rigidity and hardness, drive the response to the applied pressure. During the first stages of the mechanical pressing, oil from seed bodies flows and saturates the interconnected void of the porous medium. The compressibility of each volume, or better the overall compressibility of the press cake, is a main parameter driving the final expression step, commonly called consolidation. With the aim of testing the role of cake structure properties on ultrasound efficiency, the liquid‐to‐solid mass ratio of the press cake under study as well as the solid size of the dispersed phase were evaluated for their role on press cake compressibility. Hence three sets of experiments were set up: sonication of the raw press cake, sonication of water‐diluted press cake and sonication of milled press cake. A preliminary evaluation of the best dilution conditions was carried out by adding to the cake different amounts of water (20%, 40% and 60% w/v) under the ultrasound condition equal to 500 W, 20 kHz, 152 μm and 2 min. Best oil extraction was found with a 40:100 (w/v) water‐to‐substrate ratio. This dilution was therefore chosen for further trials.

**Figure 2:**
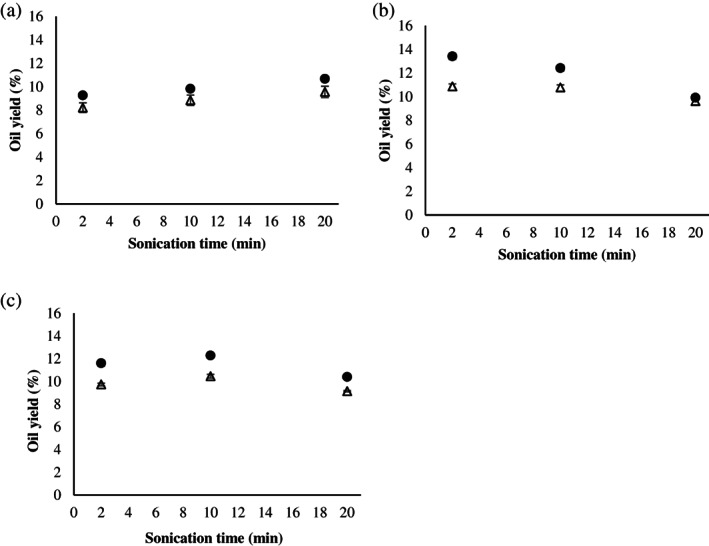
Oil extraction yield for raw (a), soaked (b) and milled (c) press cakes at different sonication amplitudes (80 μm (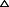) and 152 μm (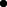)) and sonication duration (2, 10, 20 min).

The results show an increase in the oil extraction efficiency with respect to the unsonicated hempseed press cake. When ultrasound amplitude increases from 80 to 152 μm, a higher number of cavities are induced within the matrix since the number of compression and rarefaction cycles of ultrasonic waves increases, thus improving the extraction process with a significant increase in oil recovery. A significant variation in oil recovery from ultrasonicated samples is clear for both ultrasound amplitude conditions. Extraction efficiency increases linearly with ultrasonication time up to an oil yield improvement equal to 9.56% ± 0.07% and 10.66% ± 0.07% at an amplitude of 80 and 152 μm, respectively (Fig. 2(a)). Data are in agreement with previous findings:^12^, ^14^, ^26^, ^27^: ultrasound physical stresses disrupt the cell wall, increase the permeability of the cell wall and intensify the rate of mass transfer.

Even better results are obtained when the press cake is soaked with water (Fig. 2(b)). A further increase in oil recovery with respect to the unsonicated press cake is clear after 2 min ultrasound processing, for both tested amplitude levels (10.87% ± 0.17% and 13.41% ± 0.45% at 80 μm and 152 μm, respectively). Water enhances tissue swelling, enhances the hydrolytic reactions necessary for the recovery process^28^, ^29^ and facilitates diffusion and mobility of oil: when water soaks the press cake, a change occurs in the oil‐to‐water volume ratio within the emulsion phase of the dispersion that facilitates diffusion phenomena. However, the oil extraction rate decreases progressively after a certain sonication time until reaching 9.63 ± 0.17 and 9.92 ± 0.14 at 80 μm and 152 μm, respectively. This behaviour suggests that ultrasonic waves have more effect in the first stage of the extraction. Some authors ascribe similar results to the weakening of the cavitation effect with a lower oil diffusion.^30^, ^31^ The reduction in extraction efficiency with prolonged sonication time can tentatively be ascribed to the loss of solvent by evaporation, which can directly affect the loss of mass transfer.^32^

Alternatively, press cake structure was modified by comminution, using a mill operating at 15000 × *g* for 5 min before ultrasound treatments As shown in Fig. 2(c), the yield increased from 9.73% to 10.46% and from 11.60% to 12.30 after 10 min of extraction sonication under 80 and 152 μm amplitude conditions, respectively, and then a final destabilization occurred with increase in extraction time. For the process under study, the efficient extraction period for achieving maximum yield of hempseed oil was about 10 min. Comminution disrupts cellular tissue, cuts fibres and releases liquid. An extensive comminution may increase the amount of fines that can block the capillary passages of the cake and thus impair draining. Moreover, when an unstable dispersion is subjected to a mechanical action, an increase in the effective surface area of the oil droplets occurs and therefore mass transport is facilitated until a turnaround is observed, due to the previously described compaction of the solid phase. This hypothesis is supported by other authors.^33^

**Figure 3:**
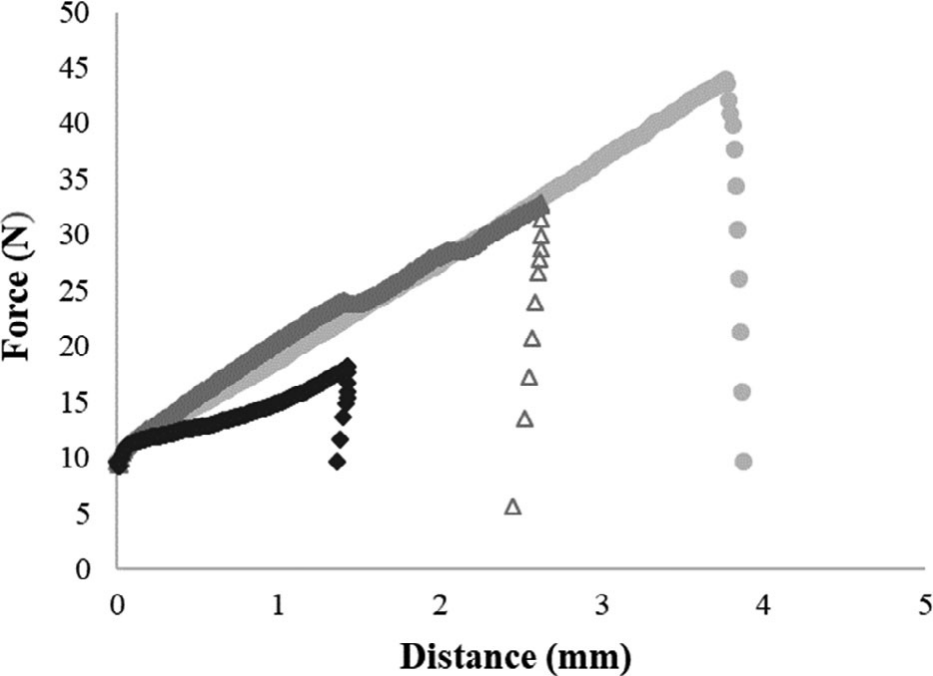
Compression–extrusion profiles for raw press cake ( 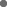), milled press cake (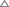) and soaked press cake (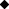) treated with ultrasound (152 μm, 2 min).

Ultrasound efficiency in the oil extraction yield therefore seems to be affected by the compressibility of the press cake. In this study, six press cake samples were tested for mechanical properties in order to obtain a quantitative index for the apparent compressibility: the milled press cake and press cake imbibed with 40% (w/v) of water, ultrasound or less treated, and raw non‐pretreated press cake. The best ultrasonication conditions in terms of oil yield were selected and kept constant for all the samples; namely, 500 W, 20 kHz and 152 μm amplitude and treatment time at 2 min were considered. Samples were placed in a square‐section test cell and a loose‐fitting plunger pressed the sample until extrusion through an extrusion plate located in the base of the cell occurred. All samples were compressed up to 40% of their original height. Figure 3 shows, as an example, three typical compression runs, on force/distance coordinates, for raw press cake, soaked press cake and milled press cake treated with ultrasound (152 μm, 2 min).

The compression and extrusion method by means of the Ottawa cell is suitable for products that are easy to extrude. Hardness was measured in the first stage of the compression and describes a product that displays substantial resistance to deformation. Generally, the higher the force required to extrude, the harder was the sample. The method proved useful for describing different textures of the bulk sample analysed.

The results suggest that the different press cake treatments on the unsonicated sample influenced the hardness and compression work values. On the other hand, the soaked press cake with ultrasonication operated at amplitudes of 152 μm showed hardness values significantly different with respect to the raw and milled press cake treated with ultrasound.

Ultrasonication, as expected, dramatically lowered the cake resistance to oil expression in both pretreated and non‐pretreated samples. The apparent compressibility of the materials after ultrasonication, quantified as the maximum compression force, was lowered to 43.65 ± 6.11 N from 150.41 ± 5.65 N in the case of the raw non‐pretreated press cake, to 18.61 ± 2.26 N from 93.74 ± 4.84 N in the case of the water‐soaked press cake and finally to 36.89 ± 8.97 N from 82.59 ± 2.17 N for the milled press cake, respectively (Table 1). The lowest apparent compressibility was expressed by the water‐diluted press cake sample. Barteri *et al*.^34^ have shown how ultrasound processing can modify the structural material though its physical and chemical effects, leading to structural changes at the macro level. At this stage of the studies on cake compressibility upon sonication, it has been verified that for hempseed oil extraction the particle size and packing, together with dilution of the oil‐in‐water emulsion soaking the solid fraction of the cake, have a role in ultrasound interaction with the semi‐exhausted press cake. Placing the ultrasound unit before the final compaction zone of the screw press is useful for the purpose of increasing the extraction yield, provided the compressibility of the cake is properly managed to enhance mass transfer.

**Table 1:** Hardness and compression work of different press cakes

Sample	Unsonicated		Ultrasound amplitude 152 μm	
	Hardness (N)	Compression work (N mm)	Hardness (N)	Compression work (N mm)
Raw press cake	150.41 ± 5.65a	140.17 ± 5.06a	43.65 ± 4.32a	59.71 ± 2.53a
Milled press cake	82.59 ± 2.17b	75.58 ± 4.70b	36.89 ± 8.97a	45.48 ± 2.87b
Soaked press cake	93.74 ± 4.84c	138.29 ± 5.15c	18.61 ± 2.76b	5.19 ± 2.10c

Values were means ± SD (*n* = 3). In each column, different letters indicate significant differences (*P*‐value < 0.05).

### Antioxidant activity of the press cake oil

In order to evaluate the impact of ultrasound extraction on the quality parameters of the expressed oil, antioxidant activity assays were performed. It is well known that phenolic compounds play a main role in oil shelf‐life.^35^ There are many methods for assessing antioxidant properties, but the DPPH assay is the simplest and most accurate method and it is commonly used to evaluate oils. The decay of the radical caused by the presence of antioxidant in the sample is monitored by the decolorization at 517 nm. The DPPH free radical scavenging of hemp oil for the different treatments was therefore evaluated. Moreover, a standard calibration curve (Trolox molecule, which is a well‐known antioxidant, was used as the standard compound for calibration curves (*R* ^2^ = 0.95) was prepared using Trolox solution and, consequently, the data were expressed as Trolox equivalent antioxidant capacity (mmol L^−1^ Trolox mL^−1^ oil) (Table 2).

**Table 2:** Antioxidant activity of press cake oil reported as Trolox equivalents antioxidant capacity (mmol mL^−1^Trolox mL^−1^ oil)

Sonication time (min)	US amplitude 80 μm			US amplitude 152 μm		
	Raw press cake	Soaked cake	Milled cake	Raw press cake	Soaked cake	Milled cake
2	22.0 ± 0.31a	27.8 ± 0.54a	24.7 ± 0.05a	22.3 ± 0.27a	25.8 ± 0.99a	24.5 ± 0.07a
10	22.7 ± 0.07b	26.0 ± 0.04b	23.1 ± 0.35b	22.0 ± 0.09b	27.1 ± 0.48b	22.2 ± 0.13b
20	25.2 ± 0.23c	23.2 ± 0.71c	21.1 ± 0.49c	25.2 ± 0.09c	22.2 ± 0.13c	20.8 ± 0.34c

Values are means ± SD (*n* = 3). In each column, different letters indicate significant differences (*P*‐value < 0.05).

In our study the DPPH scavenging activity of the unsonicated cake was found to be 71.7% ± 0.2% (22.30 mmol L^−1^ Trolox mL^−1^ oil), which is a very high value compared to the antioxidant capacity measured for other plant oils, including soybean (17.4% ± 3.2%), sunflower (23.8% ± 2.1%), lax (19.3% ± 2.1%).^36^ Siger *et al*.^37^ founded a similar value (76.2% ± 4.5%) for hempseed oil. In Table 3 the inhibition percentage values of DPPH are shown. The results suggest that the ultrasonication operated at amplitudes of 80 and 152 μm for 20 min on the raw press cake significantly affects antioxidant activity with respect to the untreated cake sample. Some authors reported that the extraction of antioxidant compounds increased as the duration of ultrasound treatment was extended.^15^, ^38^, ^39^ Accordingly, results in Table 3 for raw press cake showed a significant increase in antioxidant activity upon extension of the sonication from 2 up to 20 min (*P*‐values < 0.05) for both amplitudes. On the other hand, DPPH inhibition varies with press cake treatment. It is expected that UAE enables the release of soluble compounds within the press cake liquid–solid dispersion.

**Table 3:** DPPH radical scavenging activity and free fatty acid content (% oleic acid) as a function of ultrasound sonication time on the raw press cake, milled press cake and soaked press cake

Sample	Ultrasound amplitude 80 μm			Ultrasound amplitude 152 μm	
	Ultrasound time (min)	DPPH activity (% inhibition)	Free fatty acid (% oleic acid)	DPPH activity (% inhibition)	Free fatty acid (% oleic acid)
Raw press cake	2	70.6 ± 1.03a	1.16 ± 0.01a	71.6 ± 0.91a	1.18 ± 0.02a,b
	10	73.2 ± 0.23b	1.18 ± 0.05a,b	70.9 ± 0.28a	1.15 ± 0.03a
	20	81.6 ± 0.77c	1.21 ± 0.02b	81.6 ± 0.29b	1.20 ± 0.01b
					
Milled press cake	2	80.0 ± 0.17a	1.03 ± 0.01a	79.2 ± 0.22a	1.02 ± 0.01a
	10	74.3 ± 1.18b	1.15 ± 0.05b	71.5 ± 0.53b	1.12 ± 0.02b
	20	67.8 ± 1.65c	1.18 ± 0.02b	66.8 ± 1.14c	1.16 ± 0.01c
					
Soaked press cake	2	90.6 ± 1.82a	0.91 ± 0.01a	83.5 ± 3.35a	0.89 ± 0.01a
	10	84.4 ± 0.14b	1.05 ± 0.01b	88.0 ± 1.63b	1.08 ± 0.03b
	20	74.7 ± 2.40c	1.13 ± 0.02c	71.3 ± 0.44c	1.16 ± 0.02c

Values were means ± SD (*n* = 3). In each column, different letters indicate significant differences within the same sample type (*P*‐value < 0.05).

When the sample was treated in water, a statistically significant increase was found around 90% and 84% at 80 μm (27.8 and 26.0 mmol L^−1^ Trolox mL^−1^ oil) after 2 and 10 min sonication, respectively, with respect to the untreated sample. These results are due to the high affinity of polyphenol compounds with water.^40^ As previously commented for oil expression yield, a prolonged ultrasound treatment of up to 20 min is detrimental to antioxidant activity too, with a relevant decrease of about 1.24‐fold. A similar trend is shown for milled press cake: 80% ± 0.17% at 80 μm; 79.2% ± 0.22% at 152 μm at the shortest treatment time, down to 67.8% and 66.8% of activity (Table 3). This might be ascribed to oxidation phenomena due to the presence of oxygen.

### Free fatty acidity index of press cake oil

Free acidity content is related to the degree of lipolysis of triglycerides, and the keeping quality of the oil relies upon this parameter. Values presented in Table 3, which are expressed as percentage of oleic acid, were obtained by using the conventional analytical method. The initial free acidity content of the expressed oil was equal to 0.82% oleic acid. For different ultrasound regimes and press cake properties, free fatty acid values varied from 0.9% to 1.20% oleic acid. Sonication increases the oil fatty acid content. Hempseed oil is very susceptible to oxidative degradation due to the high content of polyunsaturated fatty acids. The most promising results are obtained for water‐imbibed press cake that are mildly sonicated (2 min).

### Ultrasound effect on hemp milled paste oil colour

Objective colour parameters are frequently used as an index of oil quality. In the CIELAB colour space the colour coordinates a* and b* and the psychometric index of lightness L* are defined on the press cake soaked and treated at 152 μm for 2 min, since this yielded the best results. The data are reported in Table 4.

**Table 4:** CIELAB colour parameters for raw and sonicated press cake oil

Sample	L*	a*	b*	ΔE (total colour difference)
Unsonicated raw press cake	3.74 ± 0.01a	5.82 ± 0.02a	6.29 ± 0.01a	—
Soaked cake with ultrasound 2 min	3.63 ± 0.02b	5.48 ± 0.01b	5.29 ± 0.04b	1.061

Values are means ± SD (*n* = 3). In each column, different letters indicate significant differences (*P*‐value < 0.05).

The change in b* (yellowness) value was more noticeable than the change in L* (lightness) and a* (redness and greenness) values. The decrease in the yellowness component (b*) may be due to reduction in carotenoids, resulting in oxidation or decomposition during ultrasound treatment. The loss in greenness component after ultrasound might be caused by the degradation of chlorophyll, due to the cavitation effect.

## CONCLUSIONS

This paper dealt with a preliminary study for an intensification of the conventional process of oil recovery from seeds by mechanical expression, with the aim of increasing the yield of oil expression from the seed. Complementary ultrasound technology was used as an out‐of‐line treatment carried out on an intermediate part of the expression process – the non‐exhausted press cake, partially de‐oiled, that was taken from the reactor near the extrusion die of the ‘exhausted’ cake. Results gathered in the investigations proved that the extraction yield is a function of the sonication conditions (amplitude, treatment time) and of the chemical–physical properties of the cake. The oily cake was treated with ultrasound both as raw press cake and modified by hydration or milling. After treatment, the apparent compressibility of the cake, which was instrumentally evaluated, changed and the extraction yield was consequently modified. This is well depicted in the theory of mechanical screw expression, because the properties of the solid–liquid dispersion change, as well as the mass transport phenomena in the cake, which are related to the physical state of the material to be fractionated. Under the experimental conditions adopted in this work, the optimal treatment in terms of final yield was reached for ultrasound use at a frequency 20 Hz and ultrasonic power of 500 W for short sonication times (2 min), at the highest amplitude of ultrasound tested (152 μm) and by sonicating the less compressible cake – that is, the one hydrated before ultrasound use. The parameters of chemical and sensorial objective quality of the oil were monitored in parallel with the various technological trials. Under optimal operating conditions, ultrasound combined with mechanical expression preserved oil quality in terms of antioxidant activity and free fatty acid index.

The hydration and milling treatments operated on the press cake had the sole purpose of modifying the properties of the material subjected to sonication. The indications provided have directed new research, currently under investigation, in which the chemical–physical properties of the cake are modulated through management of the pressure regime in the expression chamber. The aim is to obtain the most suitable cake for efficient ultrasound‐assisted expression, which has been verified to be efficient in intensifying the process of hempseed oil expression. Laboratory‐scale, out‐of‐line sonication studies gathered relevant data, which could be used in oil expression optimization studies from other oilseeds.
